# Editorial for the Special Issue on Advanced Micro- and Nano-Manufacturing Technologies

**DOI:** 10.3390/mi15121479

**Published:** 2024-12-08

**Authors:** Kun Li

**Affiliations:** 1 College of Mechanical and Vehicle Engineering, Chongqing University, Chongqing 400044, China; kun.li@cqu.edu.cn; 2 Chongqing Key Laboratory of High-Performance Structural Additive Manufacturing, Chongqing 400044, China; 3 State Key Laboratory of Mechanical Transmission for Advanced Equipment, Chongqing University, Chongqing 400044, China

With the continuous advancement of science and technology, micro- and nano-manufacturing technologies have become frontier fields in modern manufacturing [1,2]. These advanced manufacturing processes enable the creation of materials with special functions at the micro and nano scales and significantly improve manufacturing precision and efficiency [3]. High-energy, nano-, and micrometer-scale manufacturing processes have demonstrated great potential in various applications, particularly in optimizing surface properties of mechanical components and achieving complex performance regulation in the design and fabrication of functional and gradient materials [4,5,6,7,8].

Furthermore, functional materials developed through advanced manufacturing methods have broad application prospects in electronics, biomedical engineering, and aerospace [9,10,11,12]. Applying modeling and numerical analysis techniques provides a solid theoretical foundation for optimizing manufacturing processes, allowing engineers to predict and address potential issues during the design stage [13,14,15,16]. With the rise of smart manufacturing, the integration of advanced detection, monitoring, and control technologies further ensures the realization of efficient, precise, and adaptive production [17,18,19,20].

This Special Issue, Advanced Micro- and Nano-Manufacturing Technologies, brings together 21 original research articles and 1 review paper, covering the following topics: (1) high-energy/nano/micro advanced manufacturing processes; (2) material post-processing technologies; (3) advanced manufacturing design, simulation, and numerical analysis; and (4) advanced detection, monitoring, and intelligent control equipment and methods. These studies deepen our understanding of advanced manufacturing technologies and lay a solid foundation for the manufacturing industry’s future development. The following summarizes the key contributions and their scientific significance.

High-energy/nano/micro advanced manufacturing processes: To meet the high-precision manufacturing and assembly requirements of micro-mechanical systems, exploring and applying micro- and nanoscale advanced manufacturing processes is crucial [21]. Optimizing techniques, such as continuous casting (CC) and the plasma rotating electrode process (PREP), are key in minimizing material waste while enhancing material densification and mechanical properties. These advancements are essential for fabricating high-density, high-quality alloys. For instance, Lihao et al. [22] demonstrated the production of spherical silver alloy powders suitable for laser powder bed fusion (LPBF), while Likai et al. [23] used these optimized processes to fabricate dense high-entropy alloys like Fe_30_Mn_50_Co_10_Cr_10_ and Fe_50_Mn_30_Co_10_Cr_10_, showcasing significant improvements in material quality and performance.

In advanced epitaxial structures, Zhang et al. [24] designed an unbuffered AlGaN/GaN epitaxial structure on a SiC substrate, achieving superior crystallinity and surface morphology compared to traditional methods. This innovation improves control over growth stress and contributes to the fabrication of high-performance devices. Their study also emphasized the importance of precise control over printing parameters to optimize material properties in advanced manufacturing.

In digital light processing (DLP), Kang et al. [25] explored the effects of print layer thickness and orientation on surface wettability, contributing valuable insights for developing DLP-based microfluidic devices. Similarly, Sun et al. [26] developed a model to predict photoresist profile evolution in photolithography, considering factors such as incident light intensity and air gaps. This model enhances control over photoresist morphology, which is crucial for achieving precision in microfabrication.

This Special Issue also highlights other breakthroughs. For example, Tatiana L. Simonenko et al. [27] synthesized NiO, La_0.6_Sr_0.4_Co_0.2_Fe_0.8_O_3_-δ (LSCF), and (CeO_2_)_0.8_(Sm_2_O_3_)_0.2_ (SDC) nanoparticles using hydrothermal and citrate-ethylene glycol methods. These nanoparticles were applied in continuous micro-extrusion printing to create a functional ink structure with a three-layer (NiO-SDC)/SDC/(LSCF-SDC) configuration. Chen et al. [28] introduced a new source/drain (S/D) micro-adjustment process enabling vertical stacking of more than four nanosheet layers, pushing the boundaries of GAA technology beyond the 3 nm CMOS process. Zhang et al. [29] optimized the BCl_3_/SF_6_ inductively coupled plasma (ICP) etching process, achieving an impressive etching selectivity 41:1 for p-GaN. Lastly, Victor Lisitsyn et al. [30] summarized research on ceramic synthesis using high-energy electron flux, demonstrating that ceramics produced through this method exhibit similar properties to those created by traditional thermal methods, including enhanced luminescent characteristics.

Post-processing Techniques for Materials: Post-processing techniques are crucial in optimizing product performance and enhancing surface quality, particularly in micro- and nano-manufacturing processes. These techniques are essential for achieving the desired material properties and device performance [31]. This Special Issue features two research articles exploring innovative post-processing methods.

First, Sun et al. [32] introduced a novel method for forming a single-crystal AlN interface layer for Al_2_O_3_/AlN/GaN metal-insulator-semiconductor high electron mobility transistors (MIS-HEMTs). This method combines plasma-enhanced atomic layer deposition (PEALD) with in situ N_2_ plasma annealing (NPA), significantly improving the gate reliability of Al_2_O_3_/AlN/GaN MIS-HEMT devices. The synergy of these two techniques effectively enhances the overall device performance, providing a promising solution for high-reliability MIS-HEMT applications. Second, Zhao et al. [33] developed a new diamond/silica composite abrasive using a simplified sol–gel method and applied it to the semi-fixed polishing of sapphire wafers. Unlike traditional polishing slurries, this composite slurry contains only deionized water and no additional chemical additives, making it environmentally friendly. In addition to its environmental benefits, this composite slurry significantly improves polishing performance, with experimental results showing a 27.2% reduction in surface roughness and an 8.8% increase in material removal rate compared to pure diamond polishing.

Advanced Manufacturing Design, Simulation, and Numerical Analysis: The widespread use of computer simulation and numerical analysis in advanced manufacturing has made these technologies essential for reducing experimental costs and enabling in-depth research under complex conditions. This Special Issue presents three studies that showcase the contributions of simulation and numerical analysis to advancing manufacturing processes.

First, Zhaohuibo et al. [34] addressed challenges in SiCp/Al machining by designing a vibration-assisted grinding device (VLD). Through simulation and experimental testing, the team determined the device’s optimal working frequency and amplitude, validating its performance and demonstrating its potential to enhance grinding efficiency. Zhaobo et al. [35] used molecular dynamics (MD) simulations to study surface generation and subsurface damage. By analyzing surface morphology, mechanical response, and amorphization of different crystal orientations, they provided insights into how crystal orientation affects the severity of deformation and amorphization, enriching our understanding of material behavior under stress. Lastly, Han et al. [36] applied a particle filling method to manufacture neutron absorption gratings, introducing a pressurized filling technique to increase filling rates. Simulation studies revealed the effects of pressure, groove width, and Young’s modulus on filling rates. Based on these results, the team optimized the process, significantly improving manufacturing efficiency.

Advanced Detection, Monitoring, Intelligent Control Equipment, and Methods: In micro- and nano-manufacturing, advanced detection, monitoring, and intelligent control equipment are essential for effectively controlling production processes, detecting material properties, and ensuring product quality [37]. This Special Issue includes four research articles that explore innovations in these advanced technologies and methods.

Chen et al. [38] proposed a novel low-dropout voltage regulator (CL-LDO) for digital simulation hybrid circuits. Designed with a 180-nanometer process, this regulator eliminates the need for on-chip capacitors while maintaining steady-state performance, improving transient response during load current variations. Sun et al. [39] introduced a fully integrated adaptive on-time (AOT) control buck converter, capable of switching between pulse-width modulation (PWM) and pulse-skip modulation (PSM) based on load current variations, optimizing efficiency under light-load conditions. Fu et al. [40] proposed a method for manufacturing high-performance MEMS accelerometers using the TGV process. This approach reduces manufacturing costs while ensuring low-noise characteristics. Zha et al. [41] designed and fabricated a high diffraction efficiency silicon-glass grating with a working wavelength range of 800–2500 nm, suitable for near-infrared spectrometers.

These studies offer new design concepts and technical approaches for sensors and electronic devices in micro-nano manufacturing. Du et al. [42] also developed a fault detection device and method for fiber Bragg grating (FBG) sensor systems, enabling fast detection and localization of faults in aircraft FBG systems. Wang et al. [43] proposed a *Z*-axis incremental closed-loop control system based on machine vision in laser additive manufacturing, achieving real-time monitoring of precise coating heights.

Furthermore, this Special Issue also includes other innovative research. Andre Childs et al. [44] demonstrated how a traditional plotter-cutting machine can be adapted as an efficient desktop tool for rapidly producing steel mesh masks with a range below 250 μm and explored its application in healthcare. Zhao et al. [45] conducted engine bench tests to investigate the thermal insulation performance of exhaust pipes coated with basalt and fiberglass materials in different braiding forms (sleeve, wound, and felt). They also analyzed the impact of these materials on the emission characteristics of diesel engines.

Finally, the guest editors would like to express their sincere gratitude to all the authors for their valuable contributions to this Special Issue, which have greatly advanced the dissemination and development of cutting-edge micro- and nano-manufacturing technologies. We also thank the dedicated reviewers whose time and effort in reviewing the manuscripts have enhanced the quality of the submissions. We sincerely invite all researchers and professionals to continue submitting their work to this Special Issue. Your insightful contributions will further enrich the ongoing discussions and progress in this field.
